# Facile Green Synthesis of Titanium Dioxide Nanoparticles by Upcycling Mangosteen (*Garcinia mangostana*) Pericarp Extract

**DOI:** 10.1186/s11671-022-03678-4

**Published:** 2022-03-31

**Authors:** Eun-Young Ahn, Sang-Woo Shin, Kyeongsoon Kim, Youmie Park

**Affiliations:** 1 College of Pharmacy and Inje Institute of Pharmaceutical Sciences and Research, Inje University, 197 Inje-ro, Gimhae, Gyeongnam 50834 Republic of Korea; 2 Department of Pharmaceutical Engineering, Inje University, 197 Inje-ro, Gimhae, Gyeongnam 50834 Republic of Korea

**Keywords:** Green synthesis, Titanium dioxide, *Garcinia mangostana*, NIH3T3 cells, Anatase, Rutile

## Abstract

In the present report, green synthesis of titanium dioxide nanoparticles (TiO_2_ NPs) was performed by upcycling mangosteen (*Garcinia mangostana*) pericarp extract (methanol and ethyl acetate extracts). Field emission scanning electron microscopy images revealed an aggregated structure with a highly porous network of TiO_2_ NPs. TiO_2_ NPs synthesized with ethyl acetate extract (EtOAc-TiO_2_ NPs) exhibited more monodispersity and possessed smoother surfaces than the control TiO_2_ NPs (Con-TiO_2_ NPs) and TiO_2_ NPs synthesized with methanol extract (MeOH-TiO_2_ NPs). High-resolution X-ray diffraction patterns clearly confirmed that TiO_2_ NPs had a crystalline nature. A mixture of anatase and rutile was observed in Con-TiO_2_ NPs and MeOH-TiO_2_ NPs, while EtOAc-TiO_2_ NPs had only anatase with the smallest size (12.50 ± 1.81 nm). Ethyl acetate extract contained the highest amount of α-mangostin; thus, the surface of TiO_2_ NPs was functionalized with ethyl acetate extract. The functionalized TiO_2_ NPs synthesized with ethyl acetate extract (EtOAc-TiO_2_-αm) showed the highest 2,2-diphenyl-1-(2,4,6-trinitrophenyl)hydrazyl (DPPH) radical scavenging activity. In vitro cell viability on mouse fibroblast cells (NIH3T3) indicated that the newly synthesized TiO_2_ NPs did not show any significant cytotoxicity. Therefore, the TiO_2_ NPs in the present report have the potential to be used in cosmetic applications such as sunscreens.

**Supplementary Information:**

The online version contains supplementary material available at 10.1186/s11671-022-03678-4.

## Introduction

An increased emphasis on green chemistry has led to the application of natural products as green reductants to synthesize metal and metal oxide nanoparticles. Green synthetic strategies aim to eliminate or minimize the use of hazardous chemicals to protect our global environment. Among natural products, plant extracts have attracted researchers’ attention due to their advantages over other natural products. When metal nanoparticles are green-synthesized by plant extracts, synergistic activity can be anticipated by combining the biological activities of both materials (i.e., metal nanoparticles and plant extract) [1–4]. Furthermore, primary and/or secondary metabolites in plant extracts play a pivotal role as stabilizing (or capping) agents of nanoparticles. Unlike microorganisms as reductants, plant extracts remove the elaborate system of culturing and isolating microorganisms.

Titanium nanoparticles (TiO_2_ NPs) are considered extremely valuable nanomaterials because of their nontoxicity, high stability and photocatalytic activity [5, 6]. Thus, TiO_2_ NPs are applied in cosmetics, chemical sensing, wastewater treatment, antimicrobial applications, hydrogen production and lithium-ion batteries [5]. In particular, TiO_2_ NPs protect the skin from UV irradiation, whose characteristics expand their application widely in sunscreens. TiO_2_ NPs have a crystalline structure and mainly occur in three phases: anatase, rutile and brookite [7]. Synthetic methods of TiO_2_ NPs generally include sol–gel synthesis, hydrothermal methods, microwave methods and green synthetic strategies [6]. Currently, green synthetic strategies using plant extracts have been actively applied to produce TiO_2_ NPs: *Aloe barbadensis* [8], *Citrus limon* [9], *Trigonella foenum-graecum* [10], *Cochlospermum gossypium* [11], *Eichhornia crassipes* [12], *Artemisia haussknechtii* [13], *Echinacea purpurea* [14], *Carica papaya* [15], *Syzygium cumini* [16], and *Citrus sinensis* [17]. The diverse activity of the abovementioned TiO_2_ NPs has been reported as follows. TiO_2_ NPs that were synthesized using *Aloe barbadensis* showed profound antibiofilm activity against *Pseudomonas aeruginosa* [8]. Furthermore, lemon-based green-synthesized TiO_2_ NPs also demonstrated antibacterial activity against *Dickeya dadantii* [9], and TiO_2_ NPs synthesized with *Trigonella foenum-graecum* exhibited antimicrobial properties [10]. Anatase TiO_2_ NPs green-synthesized using *Cochlospermum gossypium* (Kondagogu gum) showed photocatalytic activity on organic dyes [11]. Interestingly, TiO_2_ NPs produced by *Eichhornia crassipes* were combined with morphogenic proteins [12]. These newly prepared TiO_2_ NPs demonstrated effective bone fusion behavior and enhanced antibacterial activity against pathogenic bacteria.

Mangosteen (*Garcinia mangostana* L., Clusiaceae) is a tropical fruit, and its pericarp is a waste. Mangosteen contains the powerful antioxidant xanthone as a major secondary metabolite that contributes to the biological activity of mangosteen extract [18]. The following xanthones have been isolated from mangosteen extract: α-mangostin, β-mangostin, γ-mangostin, gartanin, 8-deoxygartanin and garcinone E [19]. α-Mangostin has been reported to possess anticarcinogenic effects [20]. Along with xanthones, other phytochemicals, such as tannins, anthocyannins and phenolic compounds, are found [21]. In Southeast Asia, mangosteen has been used as a traditional medicine. Diverse biological activities of mangosteen have been reported, including anti-inflammatory, antioxidant, antiulcer, antibacterial, antifungal, central nervous system depressant, and anti-HIV activities [22]. In the authors’ laboratory, asymmetric dumbbell-shaped silver nanoparticles and spherical gold nanoparticles were synthesized by upcycling mangosteen pericarp waste extracts via a green strategy [23]. We found that the hydroxyl functional groups of phytochemicals from phenolic compounds, flavonoids, carbohydrates, and glycosides were considered major contributors to the reduction reaction of metal salts to nanoparticles. Mangosteen silver nanoparticles showed in vitro cytotoxicity on both A549 (a human lung cancer cell line) and NIH3T3 (a mouse fibroblast cell line) [23]. Specifically, the cytotoxicity of silver nanoparticles on A549 cells was closely associated with apoptotic cell death [23].

Very few articles have been published regarding the green synthesis of TiO_2_ NPs using mangosteen pericarp extract. In the present report, TiO_2_ NPs were synthesized via a green route by upcycling mangosteen pericarp extract. Furthermore, an ethyl acetate extract of mangosteen pericarp was used for functionalization on the surface of green-synthesized TiO_2_ NPs. α-Mangostin, a major component of ethyl acetate extract, was fully identified by mass spectrometry (ESI-QTOF-MS) with tandem mass (MS/MS) in both positive and negative ionization modes. Chromatographic, spectroscopic and microscopic methods were employed to characterize the extracts and nanoparticles, including reversed-phase high-performance liquid chromatography (RP-HPLC), UV–visible spectrophotometry, high-resolution X-ray diffraction (HR-XRD), field emission scanning electron microscopy (FE-SEM) and field emission transmission electron microscopy (FE-TEM). The hydrodynamic size and zeta potentials were also determined. 2,2-Diphenyl-1-(2,4,6-trinitrophenyl)hydrazyl (DPPH) radical scavenging activity and in vitro cell viability on NIH3T3 cells were examined to investigate possible applications of the newly synthesized TiO_2_ NPs for cosmetics such as sunscreens.

## Experimental Section

### Materials

Frozen mangosteens imported from Thailand were purchased from a local food market (Gimhae, Gyeongnam, Republic of Korea). Titanium tetraisopropoxide (TTIP) and α-mangostin were obtained from Sigma–Aldrich (St. Louis, MO, USA). Methanol, ethanol, ethyl acetate and isopropyl alcohol were purchased from Duksan (Gyeong-gi, Republic of Korea). HPLC grade water and acetonitrile were obtained from Burdick & Jackson (Ulsan, Republic of Kore). Deionized water was used to prepare aqueous solutions. All other reagents were of analytical grade and used as received.

### Instruments

Extraction was processed with a WUC-A22H sonicator (Daihan Scientific Co. Ltd., Seoul, Republic of Korea). An electric muffle furnace was used for calcination of TiO_2_ NPs (Jongro, Republic of Korea). UV–visible spectra were acquired with a Shimadzu UV-2600 spectrophotometer (Shimadzu Corporation, Kyoto, Japan). The hydrodynamic size and zeta potentials were determined with a NanoBrook 90 Plus Zeta analyzer (Brookhaven Instruments Corporation, Holtsville, NY, USA). FE-TEM images were obtained with a JEM-2100 model (JEOL Ltd., Tokyo, Japan). FE-SEM images were acquired with an S-4300 SE (HITACHI, Tokyo, Japan). The crystalline nature of the nanoparticles was examined using HR-XRD with a CuKα radiation source (λ = 0.154056 nm) (Ultima IV, Rigaku, Japan). A Synergy HT multidetection microplate reader was used to measure the absorbance of 96-well microplates (BioTek Instruments, Winooski, VT, USA).

### Preparation of the Extract

Three kinds of mangosteen pericarp extracts (methanol extract, water extract and ethyl acetate extract) were obtained according to our previous report, and a schematic procedure is presented in Additional file 1: Figure S1 [23]. To prepare each extract, the methanol extract was redispersed using a mixture of solvents (water:ethyl acetate = 1:1) in a separatory funnel. The solvent extraction procedure was performed on a funnel shaker (MMV-1000 W, EYELA, Tokyo, Japan) for 1 h and repeated three times. Upon finishing the extraction, each water and ethyl acetate fraction was collected. Water was removed by freeze-drying and ethyl acetate was removed by evaporating under reduced pressure using a rotary evaporator to produce water extract and ethyl acetate extract.

### Quantitative Analysis of α-mangostin by RP-HPLC

For a quantitative analysis of α-mangostin, RP-HPLC analysis was employed for each extract (methanol, water and ethyl acetate extracts). The Shimadzu HPLC system was composed of a pump (LC-20AT), autosampler (SIL-20AC), detector (SPD-M20A), column oven (CTO-20A) and degassing unit (DGU-20A5R) (Shimadzu Corporation, Kyoto, Japan). The detection wavelength was set at 316 ± 8 nm. An Aquasil C18 column (150 mm length × 4.6 mm diameter, 5 μm particle size) was purchased from Thermo Scientific (Munich, Germany), and HPLC grade solvent was utilized. Gradient elution was employed with A (water) and B (methanol) at a flow rate of 1 mL/min for 30 min. The temperature of the column oven was maintained at 30 °C. The gradient elution was as follows: 0 min (50% A, 50% B), 15 min (0% A, 100% B), and 30 min (0% A, 100% B). The amount of sample that was injected was 10 μL. Each sample was syringe-filtered (0.2 μm PTFE) prior to analysis and analyzed in triplicate. To construct a calibration curve, the standard α-mangostin was prepared at concentrations of 0.4, 0.6, 0.8, 1.0, and 2.0 mM. The calibration curve showed a linear relationship with a correlation coefficient of r^2^ = 0.995.

### Green Synthesis of TiO_2_ NPs Using the Extracts

Additional file 1: Figure S2 demonstrates a schematic illustration of the synthetic process of TiO_2_ NPs. Titanium tetraisopropoxide (TTIP) was used as a precursor of nanoparticles for the synthesis. In a mixture of isopropyl alcohol (9 mL) and TTIP (100 μL), one of the following samples was added: (i) 1 mL of methanol extract (10 mg/mL in 50% methanol), (ii) a mixture of 500 µL of ethyl acetate extract (20 mg/mL in 50% ethyl acetate) and 500 µL of water, or (iii) a mixture of 900 μL of ethanol and 100 µL of water (this served as a control). Each mixture was stirred for 24 h at ambient temperature. Upon reaction completion, each resultant solution showed a colored precipitate as follows: a red–brownish precipitate for the methanol extract and a dark yellowish precipitate for the ethyl acetate extract. The control resulted in a white precipitate. The precipitate was collected by filtration using Whatman® paper. Finally, white powders of three kinds of TiO_2_ NPs were obtained by calcination at 600 °C for 4 h. Each kind of calcinated TiO_2_ NPs was labeled as follows: MeOH-TiO_2_ NPs (methanol extract was used), EtOAc-TiO_2_ NPs (ethyl acetate extract was used) and Con-TiO_2_ NPs (ethanol was used). Then, the shape, size and crystallinity of the TiO_2_ NPs were further characterized by using analytical methods.

### Functionalization of TiO_2_ NPs with Ethyl Acetate Extract

Three kinds of calcinated TiO_2_ NPs (MeOH-TiO_2_ NPs, EtOAc-TiO_2_ NPs and Con-TiO_2_ NPs), which were synthesized in the previous section, were subjected to functionalization with the extract. Ethyl acetate extract had the highest α-mangostin concentration among the extracts; thus, we selected ethyl acetate extract for functionalization of each kind of calcinated TiO_2_ NPs. The three kinds of TiO_2_ NPs were redispersed in ethanol at a final concentration of 1 mg/mL under sonication for 30 min at 25 °C. Next, each nanoparticle colloidal solution in ethanol (30 mL) was stirred at 1,200 rpm upon the addition of ethyl acetate extract (10 mg/mL, 0.3 mL). After 24 h of stirring at ambient temperature, a centrifugation process was performed at 14,593 rcf at ambient temperature for 30 min. Then, the pellet was pooled, recovered and washed with ethanol (1 mL) twice. The drying process was performed in a vacuum oven (30 °C). Finally, the three kinds of dried powder were labeled ‘α-mangostin functionalized TiO_2_ NPs’. These were labeled MeOH-TiO_2_-αm, EtOAc-TiO_2_-αm, and Con-TiO_2_-αm.

### Characterization of α-mangostin in Ethyl Acetate Extract by ESI-QTOF-MS with Tandem Mass Technique (MS/MS)

The major component of α-mangostin in ethyl acetate extract was fully characterized by using Triple TOF 5600 + (AB SCIEX, Framingham, MA, USA). Ethyl acetate extract was analyzed by both full mass scan and tandem mass (MS/MS) with a direct injection. Electrospray ionization (ESI) was used as the ionization source with a full mass scan range of *m/z* 100 ~ 2,000. MS/MS scan range was *m/z* 30 ~ 2,000. Both positive and negative ionization modes were applied and the corresponding spectra were acquired according to the following instrumental conditions: pressure of ion source 1 (nebulizing gas), ion source 2 (heating gas) and curtain gas were 50 psi, 50 psi and 25 psi, respectively. Desolvation temperature was set at 500 °C. Ionspray voltage floating was 5.5 kV for positive ionization mode and 4.5 kV for negative ionization mode. Collision energy was 35 ± 10 V for positive ionization and -35 ± 10 V for negative ionization mode. Nitrogen was used as the collision gas.

### Assessment of Antioxidant Activity

DPPH radical scavenging activity was examined to assess the antioxidant activity according to our previous report [24]. Briefly, ethyl acetate extract was prepared at concentrations of 0.05 mg/mL, 0.1 mg/mL, and 0.2 mg/mL in ethyl acetate. Butylated hydroxytoluene (BHT) in ethanol was used as a positive control at concentrations of 0.125 mg/mL, 0.25 mg/mL, 0.5 mg/mL, and 1.0 mg/mL. Each sample (100 μL) was pipetted onto a 96-well plate, and DPPH solution (0.2 mM in ethanol, 100 μL) was added to each well. Incubation of the reaction mixture was performed for 30 min at ambient temperature. Then, the absorbance at 517 nm was measured using a microplate reader. The DPPH assay was performed in triplicate, and ethanol was used as a negative control.

The procedure of DPPH radical scavenging activity for the functionalized TiO_2_ NPs (MeOH-TiO_2_-αm, EtOAc-TiO_2_-αm, and Con-TiO_2_-αm) was slightly changed from the above method and performed as follows. One hundred microliters of each kind of the functionalized TiO_2_ NPs (5 mg/mL in ethanol) was mixed with DPPH solution (0.2 mM in ethanol, 100 μL) and reacted for 30 min at ambient temperature. Then, centrifugation was conducted at 13,844 rcf for 30 min. After centrifugation, the supernatant was pooled and utilized to measure the absorbance at 517 nm to assess the DPPH free radical scavenging activity. The DPPH assay was performed in triplicate.

### Assessment of Cell Viability

The MTT assay was applied to examine the in vitro cell viability of all TiO_2_ NPs on NIH3T3 cells according to our previous report [24]. Six samples were subjected to cell viability: MeOH-TiO_2_ NPs, EtOAc-TiO_2_ NPs, Con-TiO_2_ NPs, MeOH-TiO_2_-αm, EtOAc-TiO_2_-αm, and Con-TiO_2_-αm. Each nanoparticle sample was prepared at a concentration of 5 mg/mL in ethanol. The cells were seeded at a density of 1.0 × 10^4^ cells/well in 96-well plates. After seeding, incubation for 24 h was conducted in a 37 °C oven with a CO_2_ (5%) atmosphere. Each sample was treated on the cell with four different concentrations: 6.25 μg/mL, 12.5 μg/mL, 25 μg/mL and 50 μg/mL. Solvent (PBS)-treated cells were utilized as a control. Then, further incubation was performed for 24 h at 37 °C with a CO_2_ (5%) atmosphere. Next, 10 μL of MTT reagent (5 mg/mL in PBS) was added. Then, an additional incubation was conducted for 3 h. The absorbance at 570 nm was determined using a microplate reader.

## Results and Discussion

### Quantification of α-mangostin in the Extracts Using RP-HPLC Analysis

The mangosteen pericarp contains diverse metabolites, including xanthone (α-, β-, γ-mangostin), proanthocyanidin, protocatechuic acid, tannin, saponin, pectin, garcinone D and gambogic acid [25, 26]. Among those compounds, α-mangostin is one of the major constituents; therefore, we selected this compound as a marker in each extract for RP-HPLC analysis. The UV–visible spectrum of the standard α-mangostin exhibited two major absorbances at 242 nm and 316 nm (Additional file 1: Figure S3). Thus, we decided to use the absorbance at 316 nm to analyze α-mangostin. Quantification of α-mangostin was performed by using RP-HPLC to analyze the amount in methanol extract, ethyl acetate extract and water extract (Additional file 1: Figure S4). The standard α-mangostin was eluted at 16.549 min (Additional file 1: Figure S4A). The methanol extract and ethyl acetate extract contained α-mangostin, which had retention times of 16.552 min (Additional file 1: Figure S4B) and 16.538 min (Additional file 1: Figure S4C), respectively. However, the water extract did not contain any α-mangostin (Additional file 1: Figure S4D). When the same concentration (0.1%) of methanol extract and ethyl acetate extract was analyzed for α-mangostin, ethyl acetate extract (1.35 mM α-mangostin) contained 586 times higher amount than methanol extract (0.0023 mM α-mangostin) (data not shown).

### Identification of α-mangostin in Ethyl Acetate Extract by Using ESI-QTOF-MS

α-Mangostin (C_24_H_26_O_6_), the major component of ethyl acetate extract, was fully identified by using ESI-QTOF-MS in both positive and negative ionization modes. In each ionization mode, full mass scan and MS/MS scan were performed and the obtained spectra are demonstrated in Additional file 1: Figure S5 (positive ionization mode) and Additional file 1: Figure S6 (negative ionization mode).

In Additional file 1: Figure S5A, the full mass scan in the positive ionization mode showed that a protonated molecular ion of α-mangostin appeared at *m/z* 411.1801 [M + H]^+^ in ethyl acetate extract. In Additional file 1: Figure S5B, a precursor ion at *m/z* 411.1777 [M + H]^+^ was fragmented and MS/MS fragmentation patterns generated three diagnostic fragment ions at *m/z* 355.1159, *m/z* 337.1052 and *m/z* 299.0528. α-Mangostin possesses two side chains which have the same structure (-CH_2_-CH = C(CH_3_)_2_). A major fragment ion at *m/z* 355.1159 was produced by fragmentation of one side chain, and a consequent water loss generated the fragment ion at *m/z* 337.1052. The fragment ion at *m/z* 299.0528 was observed when both side chains were fragmented. This result well matched the previous report by Khaw et al. [27]. Khaw and coworkers qualitatively and quantitatively analyzed α-mangostin in different parts of mangsteen by LC-QTOF-MS. They have reported the fragmentation patterns of the protonated molecular ion [M + H]^+^ of α-mangostin as follows; *m/z* 355.1268, *m/z* 337.1161 and *m/z* 299.0627 [27]. These fragment ions well matched our results in Additional file 1: Figure S5B.

In Additional file 1: Figure S6A, the full mass scan in negative ionization mode showed that a deprotonated molecular ion of α-mangostin appeared at *m/z* 409.1655 [M-H]^−^ in ethyl acetate extract. In Additional file 1: Figure S6B, a precursor ion at *m/z* 409.1661 [M-H]^−^ was fragmented and MS/MS fragmentation patterns generated four diagnostic fragment ions at *m/z* 394.1427, *m/z* 377.1397, *m/z* 351.0880 and *m/z* 339.0875. These fragment ions were also observed by Liang et al. when they analyzed α-mangostin by LC–MS/MS [28]. Liang and coworkers have found fragment ions of deprotonated molecular ion of α-mangostin at *m/z* 394.1431, *m/z* 377.1400, *m/z* 351.0884 and *m/z* 339.0880. These ions also appeared in our results (Additional file 1: Figure S6B). The proposed fragmentation pathway of [M-H]^−^ of α-mangostin was reported by Wittenauer et al. [29]. Wittenauer and coworkers proposed four major fragment ions at *m/z* 394, *m/z* 377, *m/z* 351 and *m/z* 339. The MS/MS fragment ions of α-mangostin in ethyl acetate extract are summarized in Table S1. Based on the MS/MS fragment ions in both positive and negative ionization modes, evidently, α-mangostin was identified as the major component in ethyl acetate extract.

### UV–Visible Spectra of TiO_2_ NPs

**Fig. 1:**
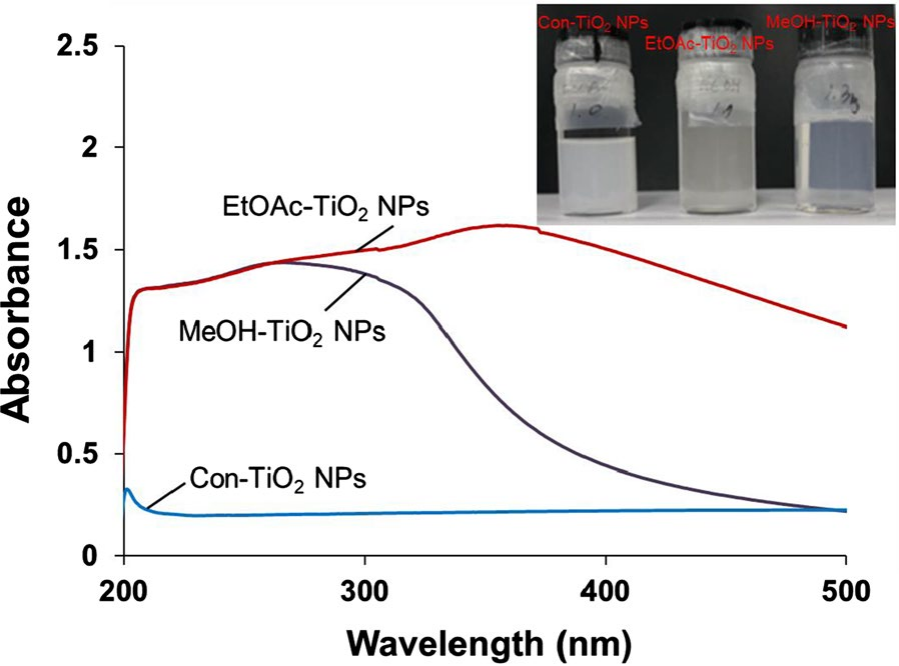
UV–visible spectra of the calcinated TiO_2_ NPs in ethanol. The black, red and blue lines indicate MeOH-TiO_2_ NPs, EtOAc-TiO_2_ NPs and Con-TiO_2_ NPs, respectively. The inset shows digital photography of each colloidal solution of Con-TiO_2_ NPs (left image), EtOAc-TiO_2_ NPs (middle image) and MeOH-TiO_2_ NPs (right image),

The calcinated TiO_2_ NPs were redispersed in ethanol, and UV–visible spectra were acquired. The UV–visible spectra provided initial confirmation of the successful synthesis of TiO_2_ NPs, and the resultant spectra are shown in Fig. 1. Con-TiO_2_ NPs (blue line) did not show any absorbance in the range of 200 ~ 500 nm. EtOAc-TiO_2_ NPs (red line) had a higher absorbance than MeOH-TiO_2_ NPs (black line). The inset in Fig. 1 shows digital photography of each colloidal solution (concentration of 0.1 mg/mL). All three kinds of nanoparticles were dispersed well in ethanol. Both EtOAc-TiO_2_ NPs and MeOH-TiO_2_ NPs were more transparent than Con-TiO_2_ NPs. It has been reported that TiO_2_ NPs as small as 20 nm are thermodynamically more stable, certainly far more transparent and provide superior SPF values for sunscreen applications [30]. The majority of TiO_2_ NPs are currently used in cosmetic applications such as sunscreen. Transparency is one of the important factors. Therefore, both EtOAc-TiO_2_ NPs and MeOH-TiO_2_ NPs are more transparent than Con-TiO_2_ NPs, suggesting the possibility of using these nanoparticles in sunscreen applications.

### FE-SEM Images

**Fig. 2:**
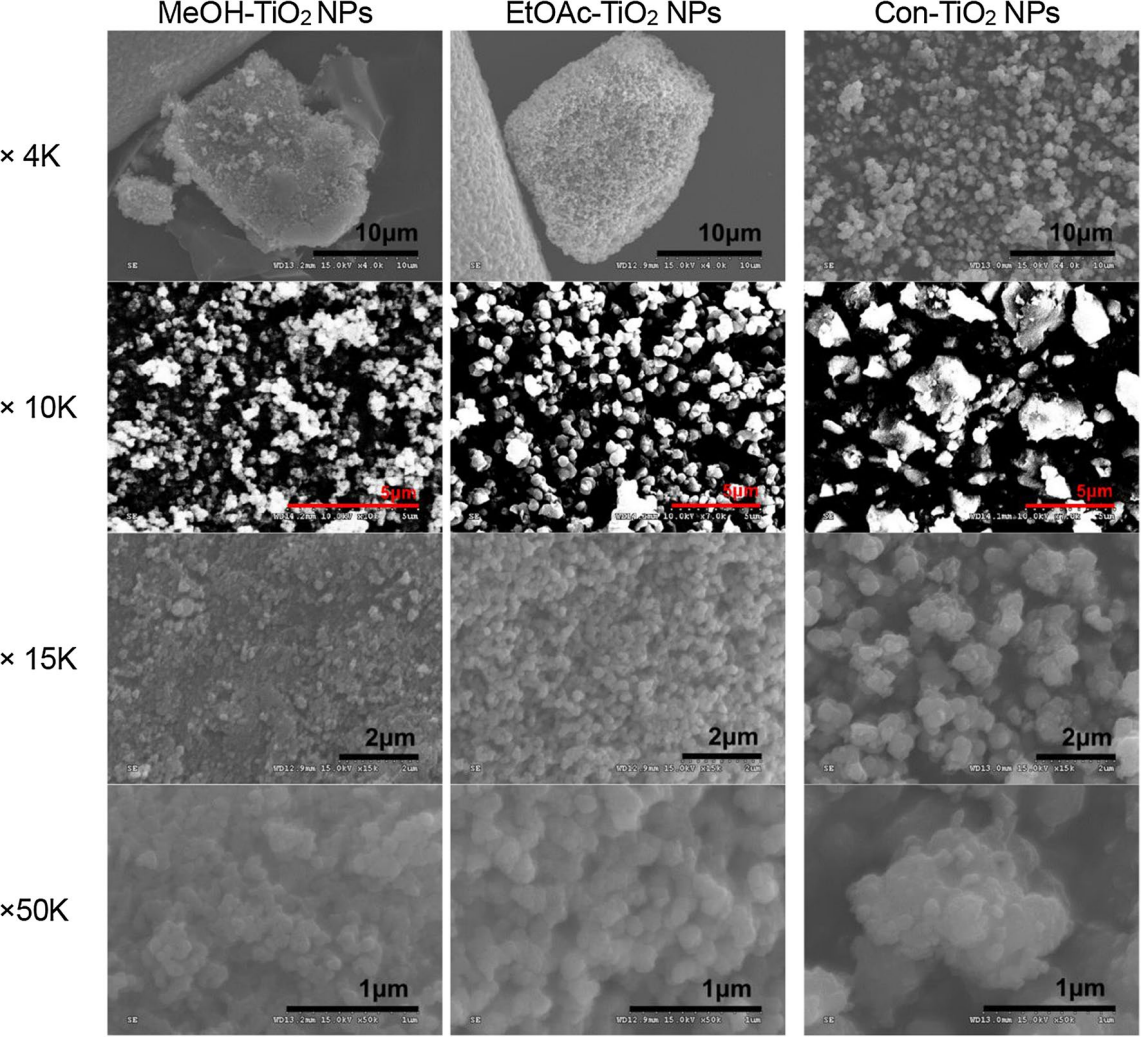
FE-SEM images. The left, middle and right columns represent MeOH-TiO_2_ NPs, EtOAc-TiO_2_ NPs and Con-TiO_2_ NPs, respectively

The surface morphology of TiO_2_ NPs was carefully examined by acquiring FE-SEM images, as shown in Fig. 2. In both × 4 K images of MeOH-TiO_2_ NPs and EtOAc-TiO_2_ NPs, the formation of aggregated (clustered) TiO_2_ NPs was clearly observed. It has been reported that aggregation is possibly due to the excess H^+^ ions of H_2_O molecules on the surface of TiO_2_ NPs. These ions reduce the repulsion between TiO_2_-TiO_2_ molecules through van der Waals forces [15]. It was also revealed that mostly spherical shapes with minor irregular shapes were synthesized in both MeOH-TiO_2_ NPs and EtOAc-TiO_2_ NPs, which was obviously observed in the enlarged images (× 10 K, × 15 K and × 50 K). Meanwhile, the shape of Con-TiO_2_ NPs was mostly irregular. Careful examination of the images (× 15 K) revealed a highly porous network of both MeOH-TiO_2_ NPs and EtOAc-TiO_2_ NPs. This interesting and unique surface morphology is common in green-synthesized TiO_2_ NPs produced by plant extracts [15–17]. The size of MeOH-TiO_2_ NPs and EtOAc-TiO_2_ NPs was much smaller than that of Con-TiO_2_ NPs. Moreover, EtOAc-TiO_2_ NPs were the most monodisperse with a smooth surface among the three kinds of TiO_2_ NPs. The monodispersity of EtOAc-TiO_2_ NPs was possibly due to the fact that only anatase was synthesized, while the other nanoparticles were mixtures of anatase and rutile. This will be discussed extensively in the section of HR-XRD patterns.

### FE-TEM Images

**Fig. 3:**
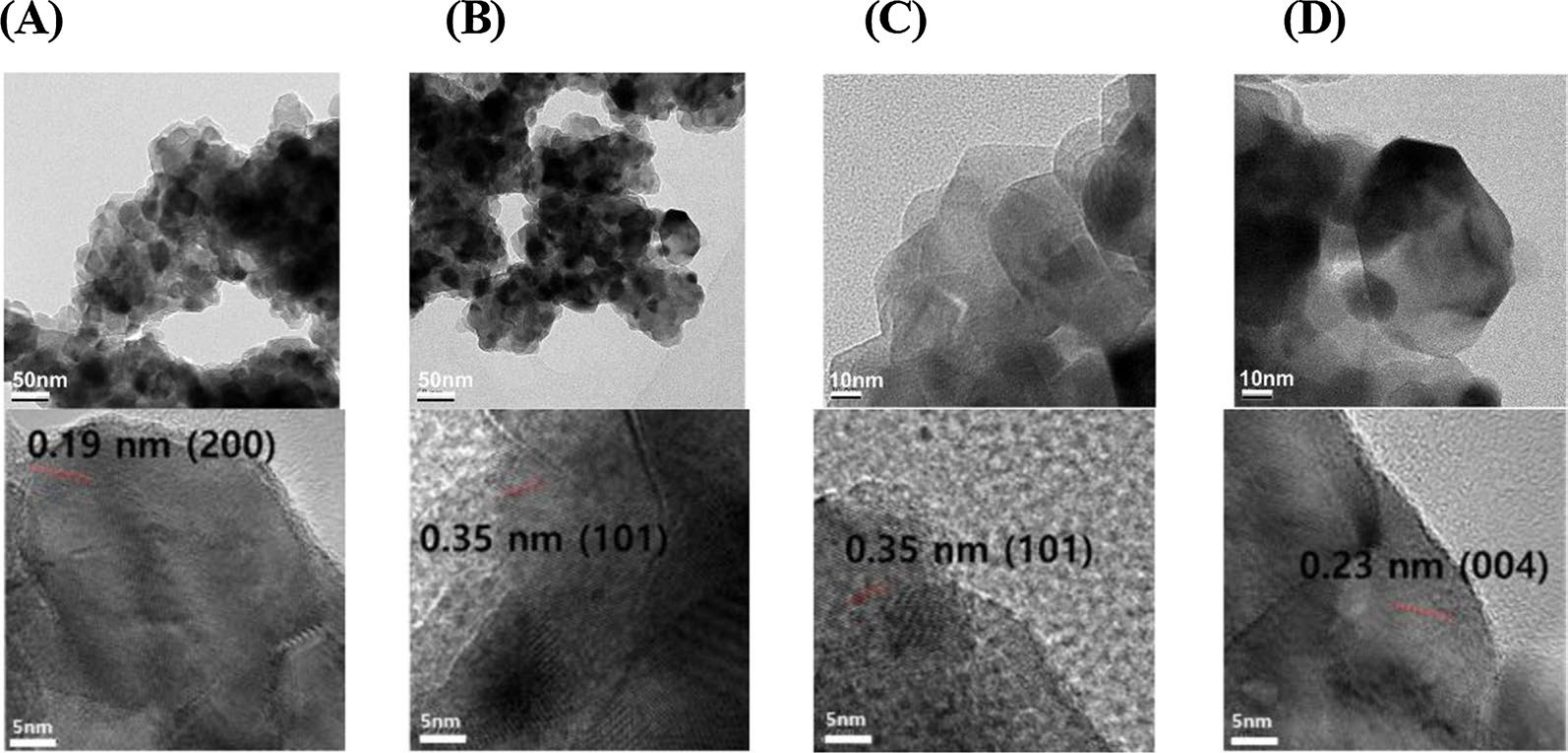
FE-TEM images of EtOAc-TiO_2_ NPs. Each enlarged picture clearly shows the distance between neighboring lattice fringes and their corresponding plane. **a** A distance of 0.19 nm with the (200) plane, **b** a distance of 0.35 nm with the (101) plane, **c** a distance of 0.35 nm with the (101) plane, and **d** a distance of 0.23 nm with the (004) plane

**Fig. 4:**
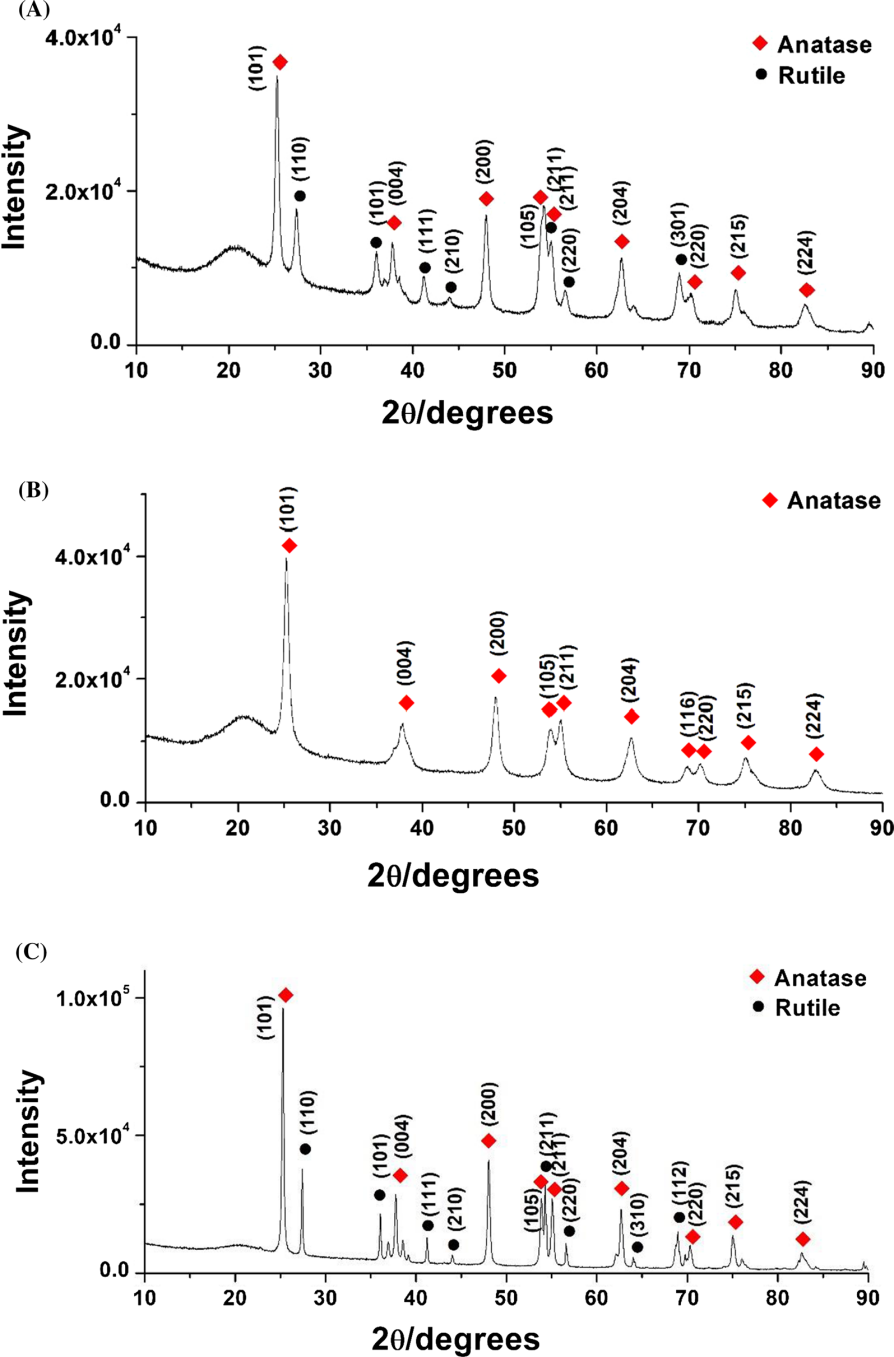
HR-XRD patterns of TiO_2_ NPs. **a** MeOH-TiO_2_ NPs, **b** EtOAc-TiO_2_ NPs, and **c** Con-TiO_2_ NPs

The FE-TEM images of EtOAc-TiO_2_ NPs are shown in Fig. 3 for further examination. Monodisperse anatase EtOAc-TiO_2_ NPs were observed with mostly spherical shapes along with irregular shapes. Each enlarged picture clearly shows the distance between neighboring lattice fringes and their corresponding plane. As shown in the HR-XRD pattern in the following section, the major plane of EtOAc-TiO_2_ NPs was the (101) plane (Fig. 4b). For the (101) plane, the distance between neighboring lattice fringes was measured as 0.35 nm (Fig. 3b and c). Along with the (101) plane, the (200) plane was clearly observed with a distance of 0.19 nm between neighboring lattice fringes (Fig. 3a). In Fig. 3d, the (004) plane was observed with a 0.23 nm distance.

### HR-XRD Patterns

TiO_2_ is a naturally occurring metal oxide. Anatase and rutile are the two most common tetragonal crystallographic polymorphs of TiO_2_ NPs [6]. Brookite is a rare orthorhombic crystalline structure [6]. In the present report, anatase and rutile TiO_2_ NPs were elucidated by HR-XRD, as shown in Fig. 4. The HR-XRD patterns were matched with the Joint Committee on Powder Diffraction Standards (No. 9015929 for anatase and No. 9015662 for rutile). The HR-XRD patterns of MeOH-TiO_2_ NPs, EtOAc-TiO_2_ NPs and Con-TiO_2_ NPs are shown in Figs. 4a–c, respectively. Careful examination of EtOAc-TiO_2_ NPs (Fig. 4b) revealed that only anatase was observed at 25.25°, 37.82°, 48.00°, 53.95°, 54.98°, 62.66°, 68.85°, 70.20°, 75.24°, and 82.80°. These peaks can be ascribed to the (101), (004), (200), (105), (211), (204), (116), (220), (215), and (224) planes, respectively. This result indicated that the crystalline nature of EtOAc-TiO_2_ NPs was anatase. Meanwhile, the HR-XRD patterns of both MeOH-TiO_2_ NPs (Fig. 4a) and Con-TiO_2_ NPs (Fig. 4c) showed a mixture of anatase and rutile. The major plane of anatase was observed as the (101) plane, and the (110) plane was found to be predominant in rutile. The HR-XRD pattern certainly confirmed that TiO_2_ NPs were successfully synthesized with crude methanol extract, ethyl acetate extract and ethanol (control). Furthermore, the size and crystalline nature were also identified. Most interestingly, ethyl acetate extract (EtOAc-TiO_2_ NPs) produced only anatase TiO_2_ NPs. The detailed peak assignment is provided in Fig. 4 and Table 1.

**Table 1:** Detailed peak assignment of HR-XRD pattern (Fig. 4) of MeOH-TiO_2_ NPs, EtOAc-TiO_2_ NPs and Con-TiO_2_ NPs

TiO_2_ NPs	diffraction planes	MeOH-TiO_2_ NPs		EtOAc-TiO_2_ NPs		Con-TiO_2_ NPs	
Phase	*hkl*	2θ (˚)	size (nm)	2θ (˚)	size (nm)	2θ (˚)	size (nm)
Anatase	(101)	25.26	18.38	25.25	12.05	25.28	35.16
	(004)	37.82	8.85	37.82	9.31	37.76	30.91
	(200)	47.99	16.22	48.00	14.92	48.04	30.83
	(105)	54.16	11.02	53.95	13.99	53.86	30.72
	(211)	55.08	17.10	54.98	13.55	55.07	27.85
	(204)	62.67	13.56	62.66	10.87	62.70	28.08
	(116)	n.d	n.d	68.85	13.24	n.d	n.d
	(220)	70.04	15.84	70.20	12.12	70.32	26.12
Average			14.42 ± 3.45		12.50 ± 1.81		29.95 ± 2.94
Rutile	(110)	27.38	18.79	n.d	n.d	27.41	51.85
	(101)	36.04	16.14	n.d	n.d	36.05	46.25
	(111)	41.22	22.87	n.d	n.d	41.22	52.43
	(211)	n.d	n.d	n.d	n.d	54.31	35.60
	(220)	56.45	10.80	n.d	n.d	56.62	40.65
	(310)	n.d	n.d	n.d	n.d	64.03	61.96
	(301)	68.90	17.15	n.d	n.d	n.d	n.d
	(112)	n.d	n.d	n.d	n.d	69.78	39.39
Average			17.15 ± 4.38		n.d		46.88 ± 9.18

n.d.: not detected

Next, based on the XRD patterns, the Scherrer equation was applied to estimate the size of TiO_2_ NPs (Table 1). The Scherrer equation is most widely used to estimate the particle size by the combination of 2θ and full width at half maximum (FWHM) values from HR-XRD patterns [31].

D = K·λ/β·cosθ (D, particle diameter; K, Scherrer constant (0.9); λ = 0.154056 nm, wavelength of X-ray radiation; β, FWHM in radians; θ, diffraction angle).

The size of MeOH-TiO_2_ NPs was estimated to be 14.42 ± 3.45 nm for anatase and 17.15 ± 4.38 nm for rutile. In the case of EtOAc-TiO_2_ NPs, only anatase was synthesized with a size of 12.50 ± 1.81 nm. The size of Con-TiO_2_ NPs was estimated to be 29.95 ± 2.94 nm (anatase) and 46.88 ± 9.18 nm (rutile), which was larger than that of MeOH-TiO_2_ NPs and EtOAc-TiO_2_ NPs. Specifically, EtOAc-TiO_2_ NPs possessed the smallest size with the smallest standard deviation (12.50 ± 1.81 nm), showing the most monodispersity among the three kinds of TiO_2_ NPs. This result was well corroborated with FE-SEM images of EtOAc-TiO_2_ NPs, which were discussed in the previous section.

### UV–Visible and FTIR Spectra of the Functionalized TiO_2_ NPs

**Fig. 5:**
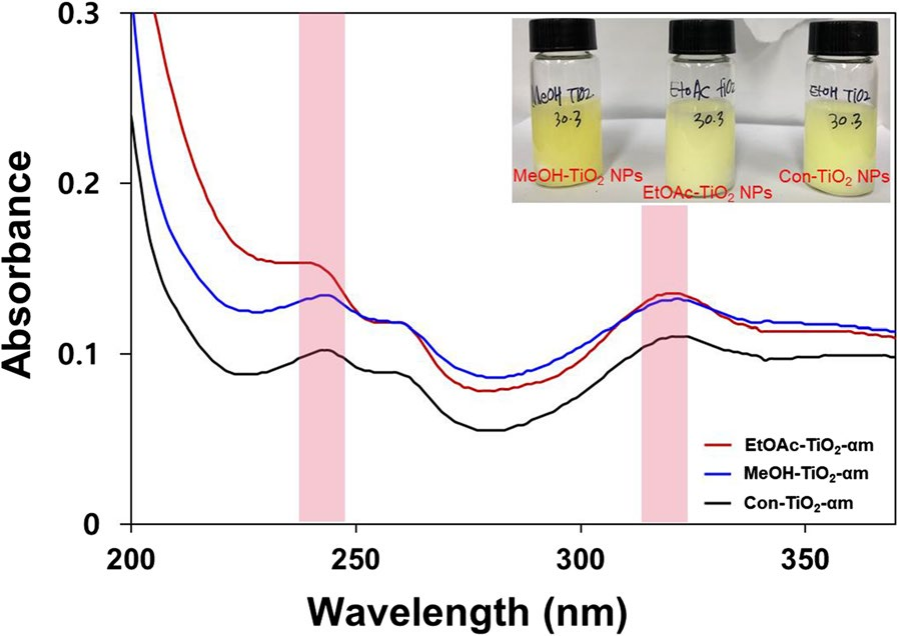
UV–visible spectra of the functionalized TiO_2_ NPs. The black, red and blue lines indicate Con-TiO_2_-αm, EtOAc-TiO_2_-αm and MeOH-TiO_2_-αm. The inset shows digital photography of each colloidal solution of MeOH-TiO_2_-αm (left image), EtOAc-TiO_2_-αm (middle image) and Con-TiO_2_-αm (right image)

UV–visible spectra of the functionalized TiO_2_ NPs (i.e., MeOH-TiO_2_-αm, EtOAc-TiO_2_-αm, and Con-TiO_2_-αm) are shown in Fig. 5. We tried two extracts (methanol extract and ethyl acetate extract) to prepare functionalized TiO_2_ NPs. When the methanol extract was utilized for functionalization, the final product was not reasonably well dispersed in solvents, which was possibly due to the high concentration of sugars and other phytochemicals. From the results of RP-HPLC analyses, ethyl acetate extract contained more α-mangostin than the methanol extract (Additional file 1: Figure S4). Thus, ethyl acetate extract was finally used for functionalization. The UV–visible spectrum of standard α-mangostin had two maximum absorbances at 242 nm and 316 nm (Additional file 1: Figure S3). UV–visible spectra of MeOH-TiO_2_-αm, EtOAc-TiO_2_-αm, and Con-TiO_2_-αm are shown in Fig. 5. All three kinds of nanoparticles displayed two maximum absorbances of α-mangostin, which are shown as two pink-colored bars in Fig. 5. This result indicated that TiO_2_ NPs functionalized with ethyl acetate extract were successfully prepared. The highest absorbance of α-mangostin at 242 nm and 316 nm was observed in the spectrum of EtOAc-TiO_2_-αm (red line), followed by MeOH-TiO_2_-αm (blue line) and Con-TiO_2_-αm (black line). The inset shows each colloidal solution of nanoparticles having a yellow color. All three kinds of nanoparticles were dispersed well in ethanol.

Next, FTIR spectra were acquired for the functionalized TiO_2_ NPs (Additional file 1: Figure S7). As shown in Additional file 1: Figure S7A, no functional groups of α-mangostin appeared in Con-TiO_2_ NPs. However, the spectra of Con-TiO_2_-αm (Additional file 1: Figure S7B), EtOAc-TiO_2_-αm (Additional file 1: Figure S7C) and MeOH-TiO_2_-αm (Additional file 1: Figure S7D) clearly showed functional groups of α-mangostin. Firstly, strong C-H stretching vibration was observed at 2,800 ~ 3,000 cm^−1^. Secondly, weak O–H stretching vibration appeared at 3,200 cm^−1^. Thirdly, strong aromatic C = C stretching vibration was observed at 1,460 cm^−1^. Finally, strong C-O stretching vibration was observed at 1,360 cm^−1^. Based on the FTIR spectra, Con-TiO_2_-αm, EtOAc-TiO_2_-αm and MeOH-TiO_2_-αm were successfully functionalized with ethyl acetate extract which included the major component of α-mangostin.

### Hydrodynamic Size, Polydispersity Index (PDI) and Zeta Potentials

As shown in Table 2, the hydrodynamic size of Con-TiO_2_ NPs was 1,115 nm with a PDI of 0.309, which decreased to 434 nm with a PDI of 0.266 in Con-TiO_2_-αm. The same observation was found for EtOAc-TiO_2_ NPs and MeOH-TiO_2_ NPs. The hydrodynamic size of MeOH-TiO_2_ NPs (757 nm) decreased to 323 nm in MeOH-TiO_2_-αm. The PDI also decreased from 0.311 to 0.250. Among the three kinds of nanoparticles, EtOAc-TiO_2_ NPs had the smallest hydrodynamic size (358 nm) with the lowest PDI (0.129). The hydrodynamic size of EtOAc-TiO_2_-αm was 147 nm with a PDI of 0.016. This result was corroborated with FE-SEM images (Fig. 2). As shown in the FE-SEM images, the EtOAc-TiO_2_ NPs were relatively monodisperse with the smallest size. Upon functionalization, EtOAc-TiO_2_-αm still maintained the smallest size. The zeta potentials of all nanoparticles were negative. When functionalization was processed, there was a tendency to increase the absolute values of zeta potential. This tendency suggests that α-mangostin in ethyl acetate extract contributed to the increase in colloidal stability of the functionalized TiO_2_ NPs.

**Table 2:** Hydrodynamic size, polydispersity index (PDI) and zeta potentials of TiO_2_ NPs

TiO_2_ NPs synthesized from	Sample	Hydrodynamic size (nm)	Polydispersity index (PDI)	Zeta potential (mV)
Methanol extract	MeOH- TiO_2_ NPs	757	0.311	-21.8
	MeOH-TiO_2_-αm	323	0.250	-31.1
Ethyl acetate				
Extract	EtOAc-TiO_2_ NPs	358	0.129	-20.5
	EtOAc-TiO_2_-αm	147	0.016	-31.0
Ethanol (control)	Con-TiO_2_ NPs	1,115	0.309	-9.1
	Con-TiO_2_-αm	434	0.266	-19.9

Upon functionalization, the hydrodynamic size decreased. Decrease in hydrodynamic size was probably due to the fact that the functionalization process with ethyl acetate extract affected the dispersion state, colloidal stability and interaction with the solvent. As previously mentioned, α-mangostin is the major component in ethyl acetate extract. Functionalization with α-mangostin decreased agglomeration and/or aggregation of nanoparticles which increased colloidal stability and dispersion state and finally resulted in the decrease in hydrodynamic size. Therefore, α-mangostin also plays a role as a stabilizer for the functionalized TiO_2_ NPs.

### DPPH Radical Scavenging Activity

**Fig. 6:**
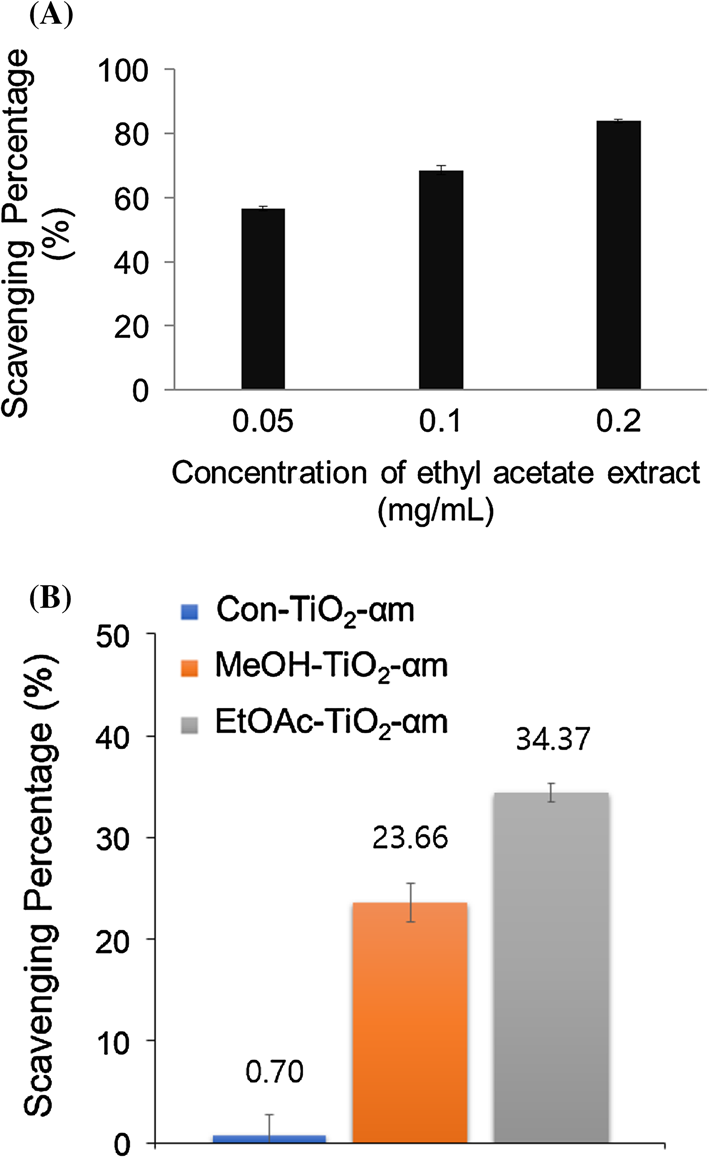
DPPH radical scavenging activity. **a** Ethyl acetate extract and **b** the functionalized TiO_2_ NPs (EtOAc-TiO_2_-αm, MeOH-TiO_2_-αm and Con-TiO_2_-αm)

Among the three extracts (i.e., methanol, ethyl acetate and water extracts), ethyl acetate extract possessed the highest amount of α-mangostin, which was demonstrated in the results of RP-HPLC chromatograms (Additional file 1: Figure S4). α-Mangostin, one of the major secondary metabolites in the mangosteen pericarp, has been known to have antioxidant activity; thus, DPPH radical scavenging activity was assessed on ethyl acetate extract (Fig. 6a). BHT was used as a positive control (data not shown). As shown in Fig. 6a, when the concentration of ethyl acetate extract was increased (0.05, 0.1 and 0.2 mg/mL), the DPPH radical scavenging activity was also increased accordingly. The scavenging activity of BHT also increased with increasing concentration (data not shown). Ethyl acetate extract showed higher DPPH radical scavenging activity than BHT. Values of IC_50_ were measured as 60 μg/mL for ethyl acetate extract and 78 μg/mL for BHT. Next, we assessed the DPPH radical activity on the three functionalized TiO_2_ NPs (Fig. 6b). Under the same concentration (2.5 mg/mL), the DPPH scavenging activity was in the order of EtOAc-TiO_2_-αm (34.37%) > MeOH-TiO_2_-αm (23.66%) > Con-TiO_2_-αm (0.70%). EtOAc-TiO_2_-αm had the highest antioxidant activity among these tested nanoparticles.

### Cell Viability on NIH3T3 Cells

**Fig. 7:**
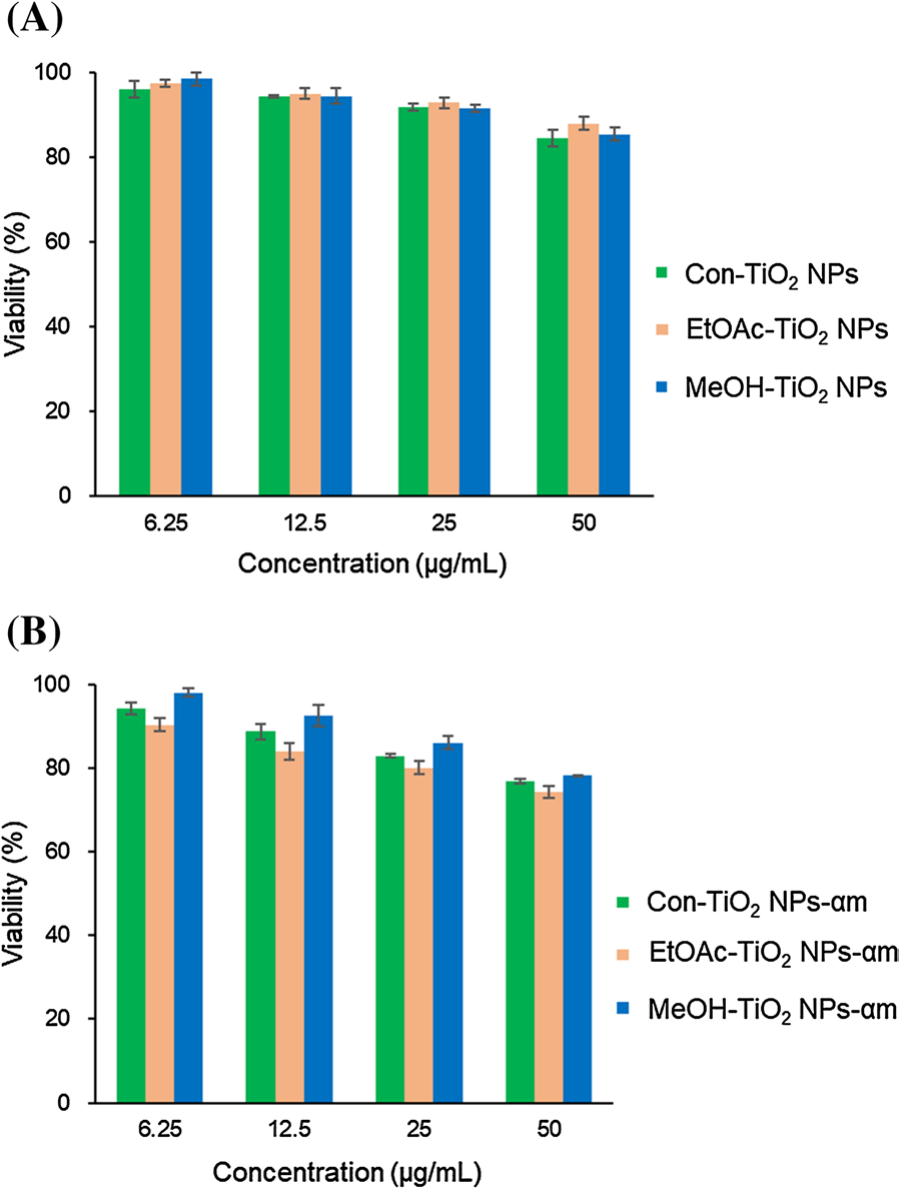
Cell viability on NIH3T3 cells. **a** TiO_2_ NPs (MeOH-TiO_2_ NPs, EtOAc-TiO_2_ NPs and Con-TiO_2_ NPs) and **b** the functionalized TiO_2_ NPs (MeOH-TiO_2_-αm, EtOAc-TiO_2_-αm and Con-TiO_2_-αm)

Cell viability was assessed in NIH3T3 cells for MeOH-TiO_2_ NPs, MeOH-TiO_2_-αm, EtOAc-TiO_2_ NPs, EtOAc-TiO_2_-αm, Con-TiO_2_ NPs and Con-TiO_2_-αm (Fig. 7). There was a dose dependency on TiO_2_ NPs (Fig. 7a) and functionalized TiO_2_ NPs (Fig. 7b) in the range of 6.25 μg/mL ~ 50 μg/mL. Upon functionalization, the cell viability was slightly lower than that of TiO_2_ NPs. When considering the highest concentration of 50 μg/mL, Con-TiO_2_ NPs had a cell viability of 84.4%, while EtOAc-TiO_2_ NPs showed a cell viability of 88.0%. After functionalization, EtOAc-TiO_2_-αm had a cell viability of 74.4%, which was the lowest among the three functionalized TiO_2_ NPs. It is most likely that α-mangostin, which was a major component in ethyl acetate extract, can cause a slight decrease in cell viability in the functionalized TiO_2_ NPs.

## Conclusion

By upcycling mangosteen pericarp extract, TiO_2_ NPs were successfully synthesized via a green strategy that is simple, nontoxic, eco-friendly, cost-effective and sustainable. TiO_2_ NPs have been used in cosmetics, in particular, as an active component in sunscreen products. EtOAc-TiO_2_ NPs possessed the smallest size (12.50 ± 1.81 nm), and only anatase was observed. Small EtOAc-TiO_2_ NPs and EtOAc-TiO_2_-αm have merits that can decrease the white appearance and enhance easy spreading on the skin. As mentioned previously, TiO_2_ NPs as small as 20 nm are thermodynamically more stable, certainly far more transparent and provide superior SPF values for sunscreen applications [30]. Furthermore, EtOAc-TiO_2_-αm had the highest DPPH radical scavenging activity (34.37%). Up to 50 μg/mL concentration in the MTT assay, the newly prepared TiO_2_ NPs displayed relatively high cell viability on NIH3T3 cells (> 70%), which suggests potential applications in sunscreen lotions. Additionally, TiO_2_ NPs were evaluated for their usage in pharmaceutical chemistry, dentistry and surgery [6]. With the combination of anticancer agents, TiO_2_ NPs offer an effective nanoplatform for drug delivery. Therefore, our future work is to apply newly synthesized TiO_2_ NPs for development as nanodrug delivery platforms.

## Supplementary Information

**Additional file 1.** **Figure S1**. Schematic illustration of extract preparation. **Figure S2**. Schematic illustration of the synthetic process of nanoparticles. **Figure S3**. UV-visible spectrum of standard α-mangostin. Maximum absorbance was observed at 198 nm, 242 nm and 316 nm. **Figure S4**. RP-HPLC analyses. (A) standard α-mangostin, (B) methanol extract, (C) ethyl acetate extract, and (D) water extract. **Figure S5**. ESI-QTOF-MS analyses of ethyl acetate extract in positive ionization mode to identify α-mangostin. (A) full mass scan, and (B) characteristic MS/MS fragmentation patterns of a protonated molecular ion at *m/z* 411.1777 [M+H]^+^ as a precursor ion. **Figure S6**. ESI-QTOF-MS analyses of ethyl acetate extract in negative ionization mode to identify α-mangostin. (A) full mass scan, and (B) characteristic MS/MS fragmentation patterns of a deprotonated molecular ion at *m/z* 409.1661 [M-H]^-^ as a precursor ion. **Table S1**. Identification of MS/MS fragmentation ions of α-mangostin in ethyl acetate extract in both positive (Figure S5B) and negative (Figure S6B) ionization modes. These fragmentation ions were well matched with the references [27–29]. **Figure S7**. FT-IR spectra. (A) Con-TiO2 NPs, (B) Con-TiO2-αm, (C) EtOAc-TiO2-αm, and (D) MeOH-TiO2-αm.

## Data Availability

All data generated or analyzed during this study are included in this published article.

## Abbreviations

Con-TiO_2_ NPs: TiO_2_ NPs synthesized with ethanol
Con-TiO_2_-αm: Functionalized Con-TiO_2_ NPs with ethyl acetate extract
EtOAc-TiO_2_ NPs: TiO_2_ NPs synthesized with ethyl acetate extract
EtOAc-TiO_2_-αm: Functionalized EtOAc-TiO_2_ NPs with ethyl acetate extract
MeOH-TiO_2_ NPs: TiO_2_ NPs synthesized with methanol extract
MeOH-TiO_2_-αm: Functionalized MeOH-TiO_2_ NPs with ethyl acetate extract
