# Supplementary material for: Facile Green Synthesis of Titanium Dioxide Nanoparticles by Upcycling Mangosteen (Garcinia mangostana) Pericarp Extract
Source: Nanoscale Res Lett. 2022 Mar 31;17:40. doi: 10.1186/s11671-022-03678-4 (PMC8971259; doi:10.1186/s11671-022-03678-4)

**Supplementary Material**

**Facile green synthesis of titanium dioxide nanoparticles by upcycling mangosteen (*Garcinia mangostana*) pericarp extract**

Eun-Young Ahn^1^, Sang-Woo Shin^2^ Kyeongsoon Kim^2^ and Youmie Park^1^

^1^College of Pharmacy and Inje Institute of Pharmaceutical Sciences and Research, Inje University, 197 Inje-ro, Gimhae, Gyeongnam 50834, Republic of Korea

^2^Department of Pharmaceutical Engineering, Inje University, 197 Inje-ro, Gimhae, Gyeongnam 50834, Republic of Korea

**Figure S1.** Schematic illustration of extract preparation.


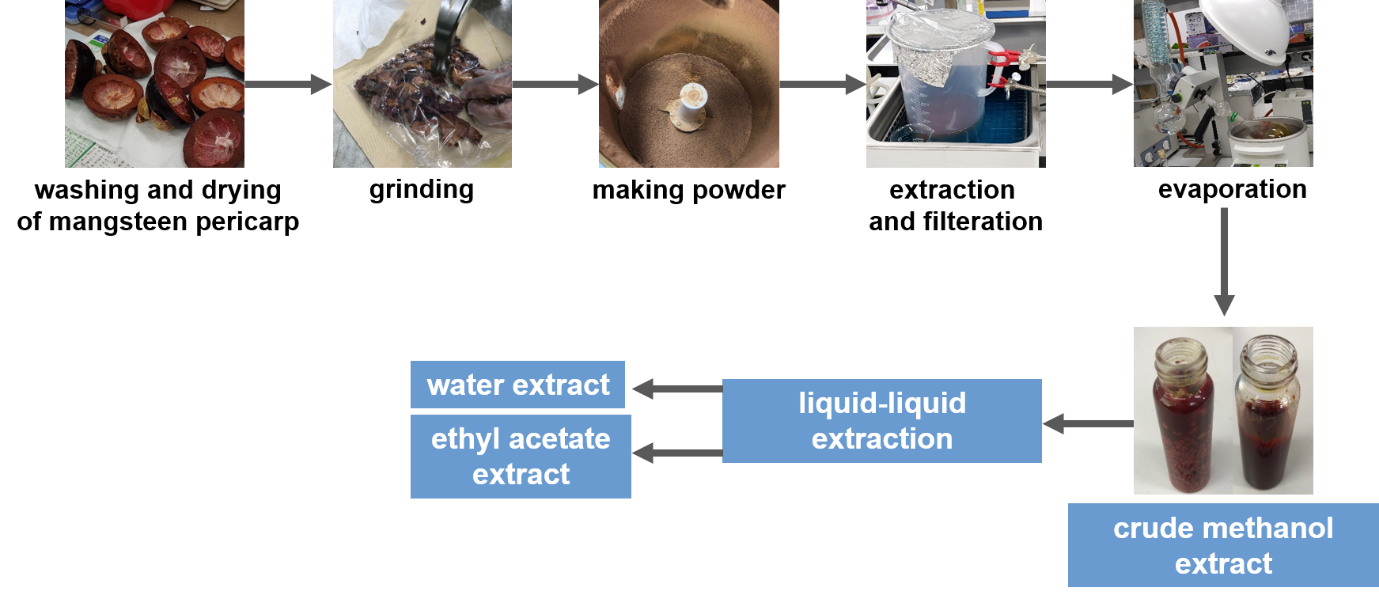


**Figure S2.** Schematic illustration of the synthetic process of nanoparticles.


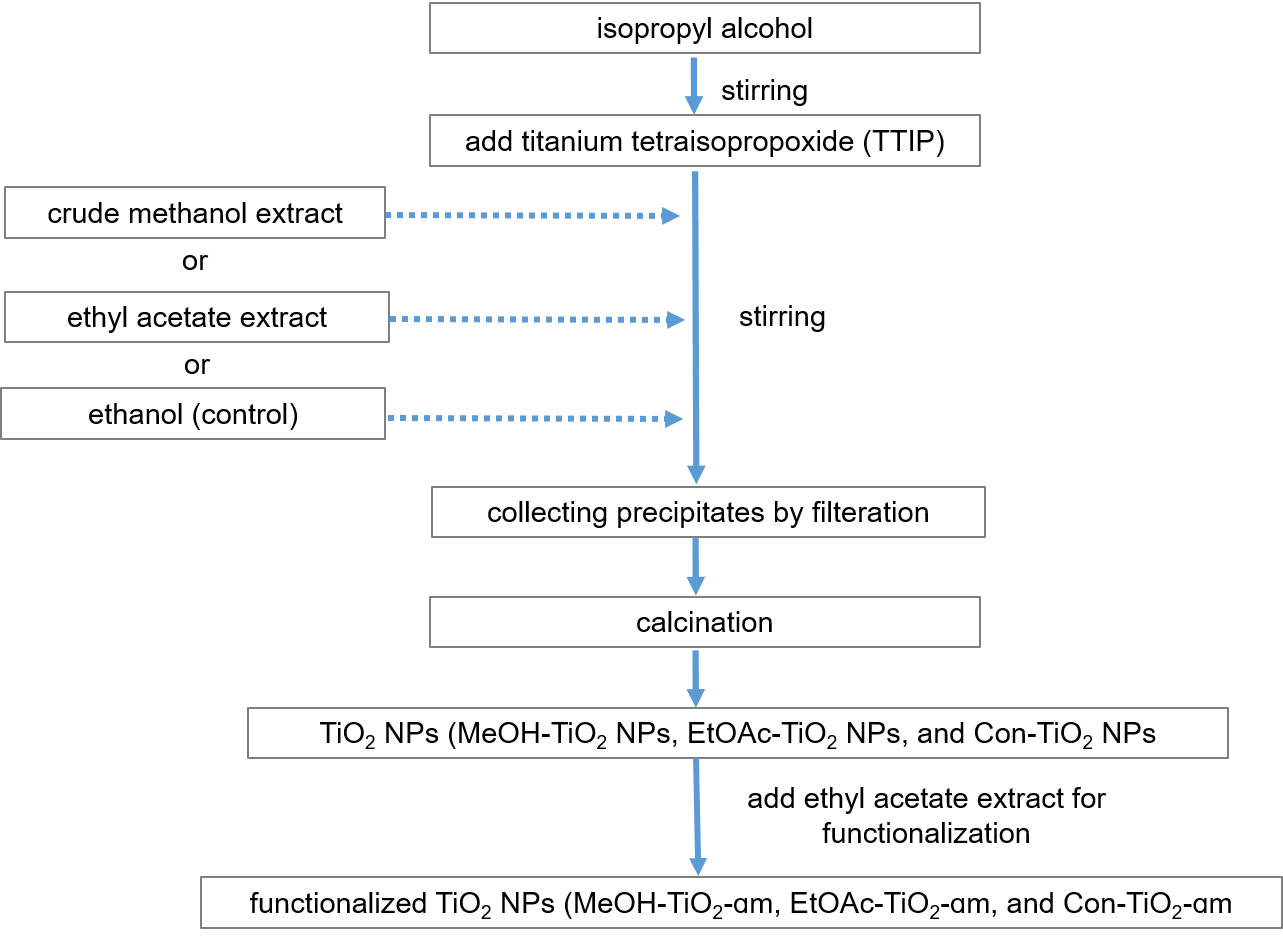


**Figure S3.** UV-visible spectrum of standard α-mangostin. Maximum absorbance was observed at 198 nm, 242 nm and 316 nm.


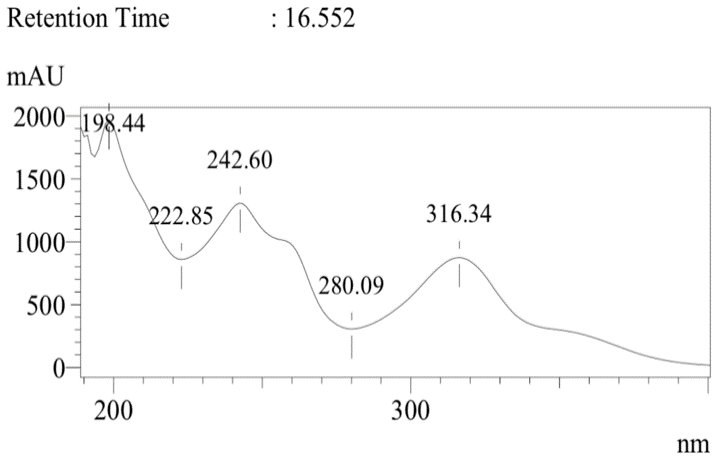


**Figure S4.** RP-HPLC analyses. (A) standard α-mangostin, (B) methanol extract, (C) ethyl acetate extract, and (D) water extract.

(A)


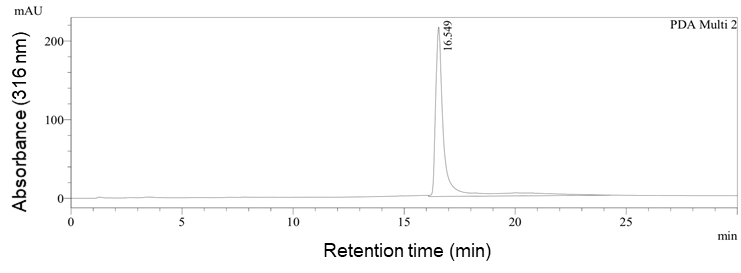


**(B)**

**
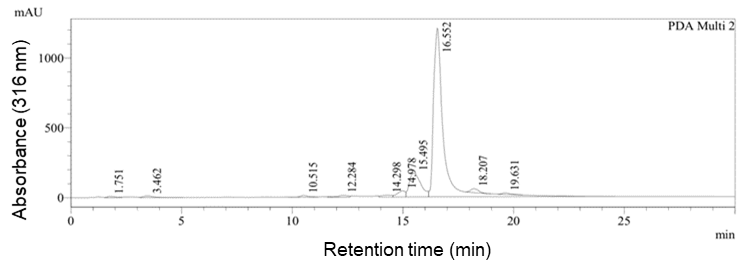
**

**(C)**

**
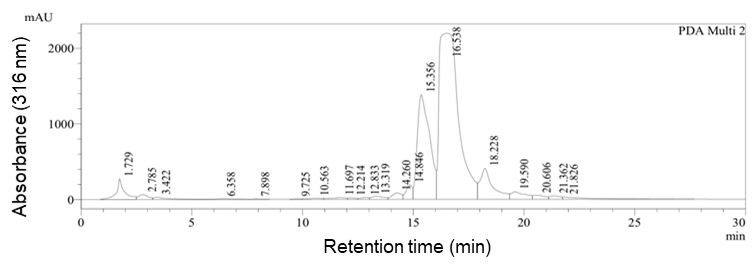
**

**(D)**

**
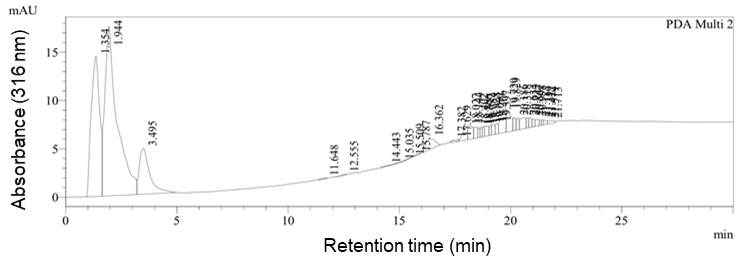
**

**Figure S5.** ESI-QTOF-MS analyses of ethyl acetate extract in positive ionization mode to identify α-mangostin. (A) full mass scan, and (B) characteristic MS/MS fragmentation patterns of a protonated molecular ion at *m/z* 411.1777 [M+H]^+^ as a precursor ion.

**(A)**

**(B)**

**Figure S6.** ESI-QTOF-MS analyses of ethyl acetate extract in negative ionization mode to identify α-mangostin. (A) full mass scan, and (B) characteristic MS/MS fragmentation patterns of a deprotonated molecular ion at *m/z* 409.1661 [M-H]^-^ as a precursor ion.

**(A)**

 **(B)**

**Table S1.** Identification of MS/MS fragmentation ions of α-mangostin in ethyl acetate extract in both positive (Figure S5B) and negative (Figure S6B) ionization modes. These fragmentation ions were well matched with the references [27-29].

| ionization mode | observed mass  (*m/z*) | proposed formula | error  (ppm) |
| --- | --- | --- | --- |
| positive  (Figure S5B) | 411.1777  (parent ion, [M+H]^+^) | C_24_H_27_O_6_ | -6 |
|  | 355.1159 | C_20_H_19_O_6_ | -4.8 |
|  | 337.1052 | C_20_H_17_O_5_ | -5.4 |
|  | 299.0528 | C_16_H_11_O_6_ | -7.2 |
| negative  (Figure S6B) | 409.1661  (parent ion, [M-H]^-^) | C_24_H_25_O_6_ | 1.2 |
|  | 394.1427 | C_23_H_22_O_6_ | - |
|  | 377.1397 | C_23_H_21_O_5_ | 0.6 |
|  | 351.0880 | C_20_H_15_O_6_ | 1.8 |
|  | 339.0875 | C_19_H_15_O_6_ | 0.3 |

**Figure S7.** FT-IR spectra. (A) Con-TiO_2_ NPs, (B) Con-TiO_2_-αm, (C) EtOAc-TiO_2_-αm, and (D) MeOH-TiO_2_-αm.


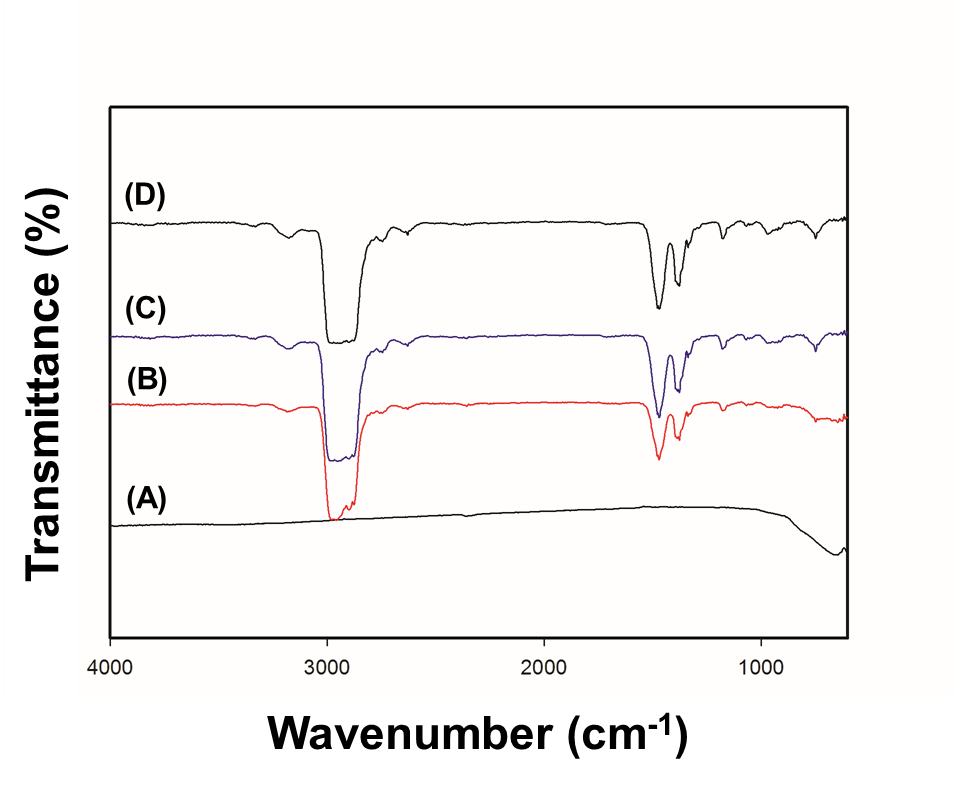

Supplement: Supplementary file 1 — Additional file 1. Figure S1. Schematic illustration of extract preparation. Figure S2. Schematic illustration of the synthetic process of nanoparticles. Figure S3. UV-visible spectrum of standard α-mangostin. Maximum absorbance was observed at 198 nm, 242 nm and 316 nm. Figure S4. RP-HPLC analyses. (A) standard α-mangostin, (B) methanol extract, (C) ethyl acetate extract, and (D) water extract. Figure S5. ESI-QTOF-MS analyses of ethyl acetate extract in positive ionization mode to identify α-mangostin. (A) full mass scan, and (B) characteristic MS/MS fragmentation patterns of a protonated molecular ion at m/z 411.1777 [M+H]+ as a precursor ion. Figure S6. ESI-QTOF-MS analyses of ethyl acetate extract in negative ionization mode to identify α-mangostin. (A) full mass scan, and (B) characteristic MS/MS fragmentation patterns of a deprotonated molecular ion at m/z 409.1661 [M-H]- as a precursor ion. Table S1. Identification of MS/MS fragmentation ions of α-mangostin in ethyl acetate extract in both positive (Figure S5B) and negative (Figure S6B) ionization modes. These fragmentation ions were well matched with the references [27–29]. Figure S7. FT-IR spectra. (A) Con-TiO2 NPs, (B) Con-TiO2-αm, (C) EtOAc-TiO2-αm, and (D) MeOH-TiO2-αm. [file 11671_2022_3678_MOESM1_ESM.docx]
